# Three-dimensional organic–inorganic hybrid sodium halide perovskite: C_4_H_12_N_2_·NaI_3_ and a hydrogen-bonded supra­molecular three-dimensional network in 3C_4_H_12_N_2_·NaI_4_·3I·H_2_O

**DOI:** 10.1107/S2053229618006885

**Published:** 2018-05-23

**Authors:** Xiao-Gang Chen, Ji-Xing Gao, Xiu-Ni Hua, Wei-Qiang Liao

**Affiliations:** a College of Chemistry and Chemical Engineering, Southeast University, Nanjing 211189, People’s Republic of China; b College of Chemistry, Nanchang University, Nanchang 330031, People’s Republic of China

**Keywords:** three-dimensional perovskite, organic–inorganic hybrid, sodium halides, iodide, hydrogen bonds, crystal structure

## Abstract

The rational selection of ligands is vitally important in the construction of new organic–inorganic hybrid three-dimensional perovskite complexes. As part of an exploration of perovskite-type materials, two new Na–I compounds based on the piperazine ligand, namely poly[piperazinediium [tri-μ-iodido-sodium]], {(C_4_H_12_N_2_)[NaI_3_]}_*n*_, **1**, and *catena*-poly[tris­(piperazinediium) [[tri­iodido­sodium]-μ-iodido] triiodide monohydrate], {(C_4_H_12_N_2_)_3_[NaI_4_]I_3_·H_2_O}_*n*_, **2**, have been synthesized by adjusting the stoichiometric ratio of sodium iodide and piperazine, and were characterized by single-crystal X-ray diffraction. In the crystal structures of **1** and **2**, each Na^I^ cation is linked to six I atoms, but the compounds show completely different configurations. In **1**, the structure includes a perovskite-like array of vertex-sharing NaI_6_ octa­hedra stretching along the direction of the three axes, and each piperazinediium dication is enclosed in the NaI_3_ perovskite cage. However, in **2**, each Na^I^ atom bridges a single I atom to form a one-dimensional linear chain, and complex inter­molecular hydrogen bonds connect these one-dimensional chains into a three-dimensional supra­molecular network.

## Abstract 2

Two new organic–inorganic hybrid sodium halides based on the piperazinediium dication have been synthesized, with C_4_H_12_N_2_·NaI_3_ presenting a three-dimensional perovskite structure and 3C_4_H_12_N_2_·NaI_4_·3I·H_2_O showing a three-dimensional hydrogen-bonded supra­molecular network.

## Introduction   

In recent decades, three-dimensional organic–inorganic hybrid perovskites have been of inter­est to researchers, not only for their remarkable structural variability and highly tunable properties, but also for their rich physical properties, such as superconductivity, ionic conductivity and ferroelectric related properties (Jin *et al.*, 2009 ▸; Saparov & Mitzi, 2016 ▸; Veldhuis *et al.*, 2016 ▸). Such hybrid perovskites have a simple generic formula of *AMX*_3_ (*A* = organic cation, *M* = metal cation and *X* = halogen anion) and the structural characteristic of corner-sharing *MX*_6_ octa­hedra. Among them, there have been a large number of reports on the halometallates of Pb^II^ and Sn^II^ ions because of their superior semiconducting properties, but related systems containing alkali metal halides are rare (Lee *et al.*, 2003 ▸; Shi *et al.*, 2017 ▸; Liao *et al.*, 2016*b* ▸; Galkowski *et al.*, 2016 ▸; Yang *et al.*, 2015 ▸; Liao *et al.*, 2016*a* ▸). To be precise, the first alkali metal halide perovskites, *RM*Cl_3_ (*R* = piperazine and *M* = K, Rb and Cs), were found less than ten years ago (Paton & Harrison, 2010 ▸). In recent years, due to the development of mol­ecular ferroelectric materials (You *et al.*, 2017 ▸; Xu *et al.*, 2017 ▸; Liao *et al.*, 2017 ▸), three-dimensional alkali metal halide perovskites have attracted the attention of researchers again. Just last year, Xiong and co-workers reported two high-*T*_c_ three-dimensional perovskite ferroelectric materials, *i.e.* [3-ammonio­pyr­roli­dinium]·RbBr_3_ and [*N*-methyl-1,4-diazo­niabi­cyclo­[2.2.2]octa­ne]·RbI_3_ (Pan *et al.*, 2017 ▸; Zhang *et al.*, 2017 ▸).

Following on from this work, we report the new three-dimensional organic–inorganic hybrid perovskite C_4_H_12_N_2_·NaI_3_ (**1**). In addition, considering that the dimensionality of three-dimensional perovskites can often be switched by alteration of the experimental conditions (*e.g.* CH_3_NH_3_·PbI_3_; Jodlowski *et al.*, 2016 ▸), we obtained a new compound, *i.e.* 3C_4_H_12_N_2_·NaI_4_·3I·H_2_O (**2**) with a peculiar one-dimensional [NaI_5_]^4−^ linear chain and a three-dimensional hydrogen-bonded supra­molecular network by adjusting the stoichiometry of piperazine and sodium iodide.

## Experimental   

### Synthesis and crystallization   

#### Synthesis of C_4_H_12_N_2_·NaI_3_, (1)   

An aqueous solution (20 ml) of sodium iodide (1.49 g, 10 mmol) was added dropwise to an equimolar ratio of piperazine (0.86 g, 10 mmol) in water (5 ml) with stirring. The solution was then filtered to remove insoluble impurities. Yellow block-shaped crystals of **1** suitable for X-ray diffraction were obtained by slow volatilization of the aqueous solution at 330 K after 2 d.

#### Synthesis of 3C_4_H_12_N_2_·NaI_4_·3I·H_2_O, (2)   

An aqueous solution (20 ml) of sodium iodide (0.75 g, 5 mmol) was added dropwise to an aqueous solution (5 ml) of piperazine (1.29 g, 15 mmol). The solution was stirred for 20 min and then filtered to remove insoluble impurities. Yellow needle-shaped crystals of **2** were obtained by slow volatilization of the aqueous solution at 330 K after 2 d.

### Refinement   

Crystal data, data collection and structure refinement details are summarized in Table 1 ▸. H atoms bonded to O atoms were located from difference Fourier maps and refined with an O—H distance restraint of 0.85 (1) Å. Other H atoms were placed in idealized positions and included as riding, with C—H = 0.97 Å (methyl­ene) or N—H = 0.89 Å. *U*_iso_(H) values were set at 1.2*U*_eq_(C,N) for methyl­ene and piperazinediium, and at 1.5*U*_eq_(O) of water H atoms.

## Results and discussion   

### Structure of C_4_H_12_N_2_·NaI_3_, (1)   

**Figure 1:**
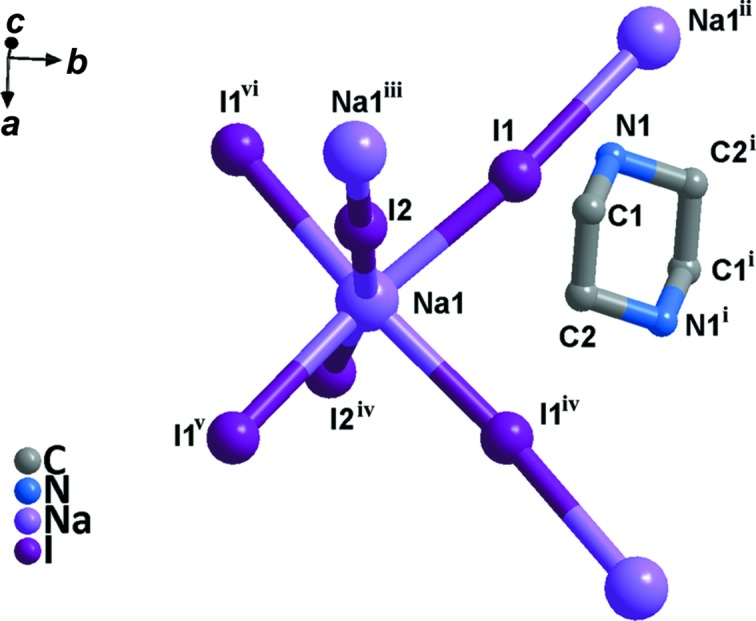
A view of the asymmetric unit in compound **1**. All H atoms have been omitted for clarity. [Symmetry codes: (i) −*x* + 1, −*y* + 2, −*z* + 1; (ii) *x* − 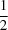, *y* + 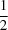, *z*; (iii) −*x* + 1, −*y* + 1, −*z* + 1; (iv) −*x* + 1, *y*, −*z* + 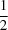; (v) *x* + 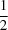, *y* − 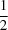, *z*; (vi) −*x* + 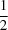, *y* − 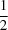, −*z* + 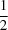.]

**Figure 2:**
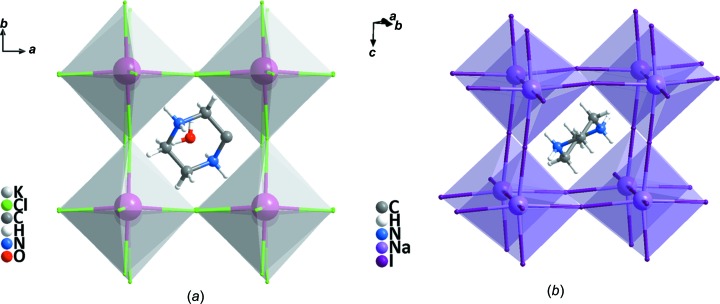
(*a*) A view of the three-dimensional perovskite cage of C_4_H_12_N_2_·KCl_3_·H_2_O. (*b*) A view of the three-dimensional perovskite cage of compound **1**.

**Figure 3:**
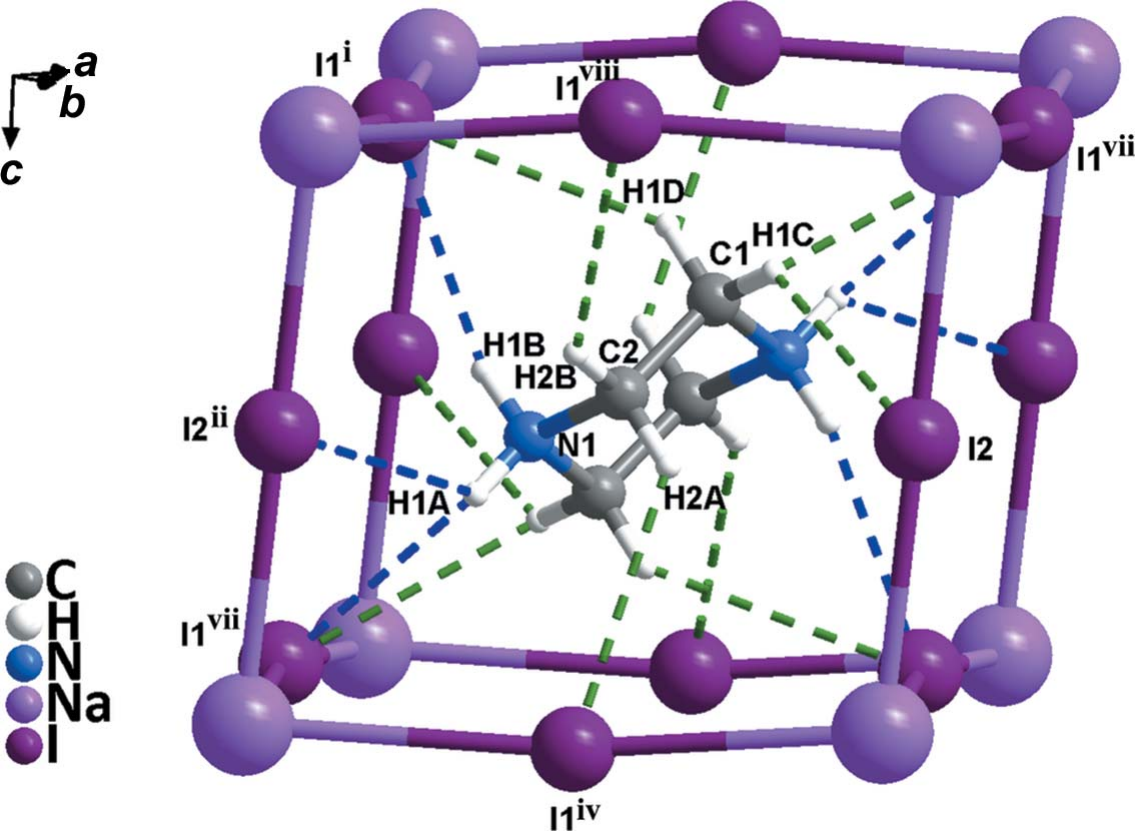
The hydrogen bonds (dashed lines) in **1** of the C and N atoms of the piperazinediium cation with the I atoms of the NaI_6_ octa­hedra. [Symmetry codes: (i) −*x* + 1, −*y* + 2, −*z* + 1; (ii) *x* − 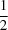, *y* + 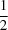, *z*; (iv) −*x* + 1, *y*, −*z* + 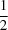; (vii) −*x* + 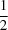, −*y* + 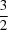, −*z* + 1; (viii) *x* + 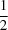, −*y* + 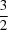, *z* + 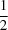.]

**Figure 4:**
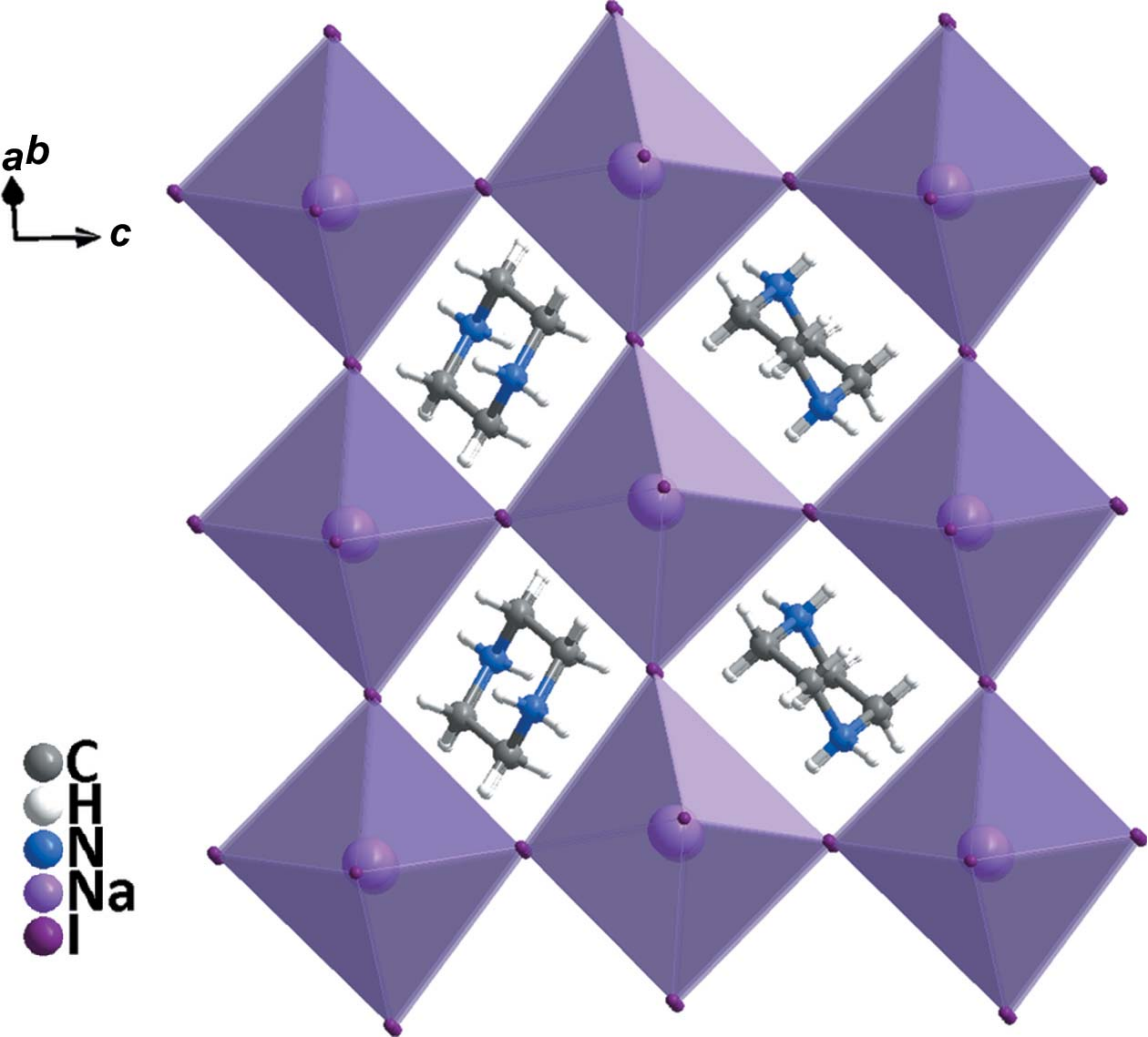
A packing view of compound **1**, showing the three-dimensional perovskite structure.

Compound **1** crystallizes in the monoclinic system (space group *C*2/*c*) and exhibits the three-dimensional perovskite framework. The asymmetry unit (Fig. 1 ▸) includes one Na^I^ cation located on a twofold axis, one half of a piperazinediium dication located about a centre of inversion and two iodide ions attached to the Na^I^ cation. As shown in Fig. 2 ▸, **1** is different from C_4_H_12_N_2_·KCl_3_·H_2_O, due to the Na—I bond length being less than that of K—Cl (Table 2 ▸); the NaI_6_ perovskite cage encloses one piperazinediium cation and prevents the entry of water mol­ecules. In addition, the H atoms on the C and N atoms of piperazinediium form weak hydrogen bonds with the I atoms in the cage, resulting in significant octa­hedral tilting (Fig. 3 ▸). According to Glazer’s 23 tilt system (Glazer, 1972 ▸, 1975 ▸), the octa­hedral tilting of compound **1** should belong to the ‘a^−^b^−^b^−^’ type. Detailed information of the C—H⋯I and N—H⋯I hydrogen bonds is given in Table 3 ▸. It can be seen from the packing diagram (Fig. 4 ▸) that the piperazinediium cations in the *ab* plane are arranged along the same direction; however, the piperazinediium cations along the *c* axis are arranged in a zigzag manner, *viz.* ‘\/\’. In summary, compound **1** has the familiar three-dimensional perovskite framework structure, where the piperazinediium cations are confined in the cavities enclosed by corner-sharing NaI_6_ octa­hedra and stabilized by C—H⋯I and N—H⋯I hydrogen bonds.

### Structure of 3C_4_H_12_N_2_·NaI_4_·3I·H_2_O, (2)   

**Figure 5:**
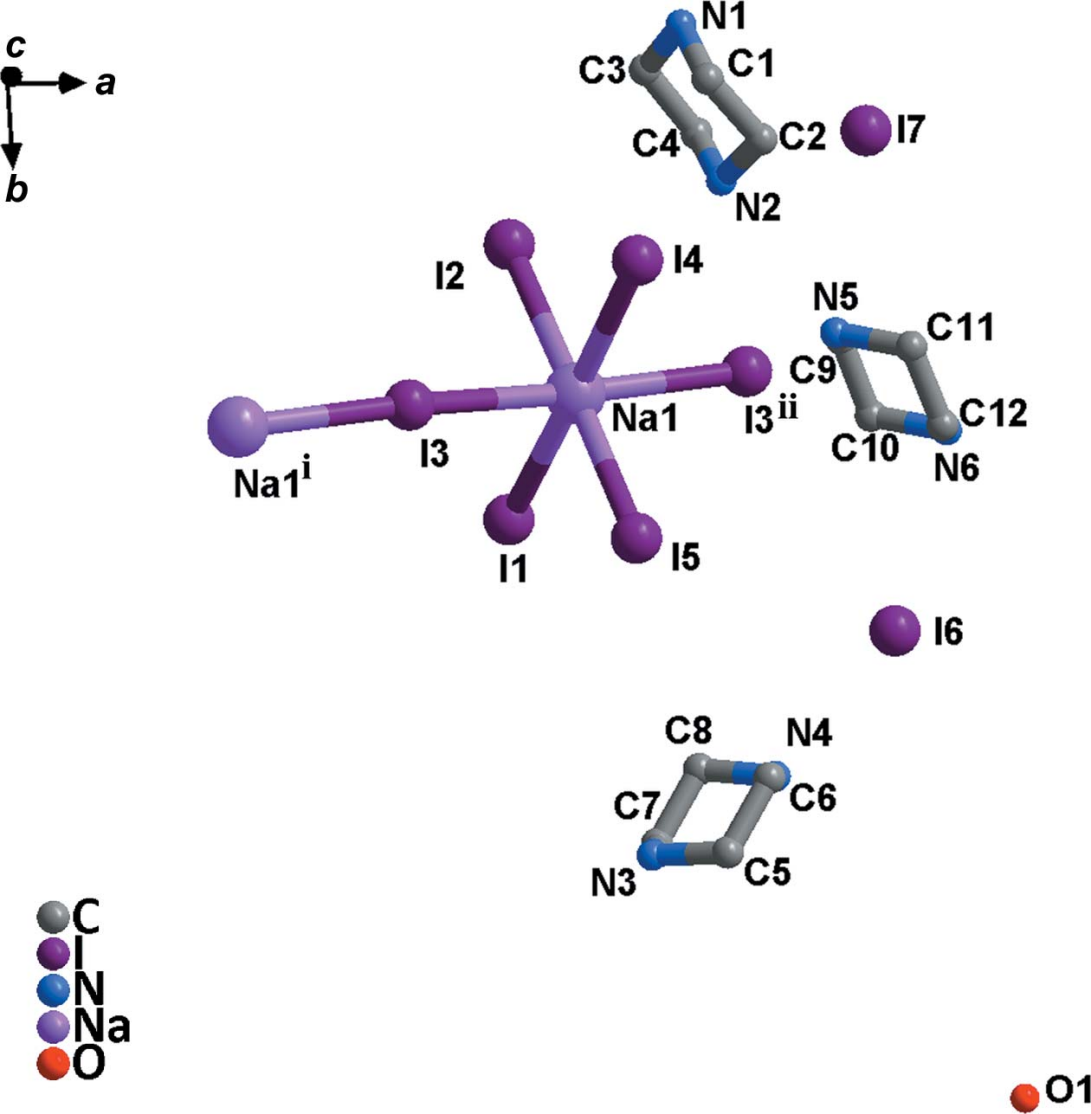
A view of the asymmetric unit in compound **2**. All H atoms have been omitted for clarity. [Symmetry codes: (i) *x* − 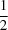, −*y* + 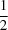, *z* − 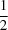; (ii) *x* + 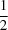, −*y* + 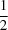, *z* + 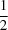.]

**Figure 6:**
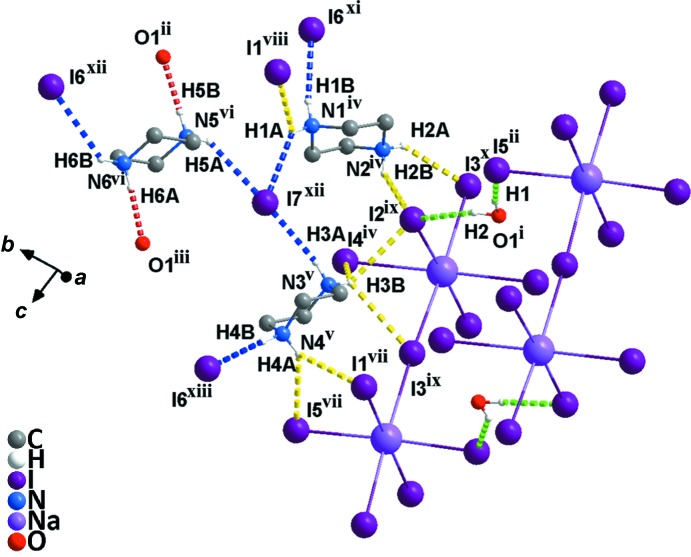
A partial view of the crystal packing of compound **2**, showing the inter­molecular N—H⋯I (blee and yellow dashed lines), N—H⋯O (red dashed lines) and O—H⋯I (green dashed lines) hydrogen bonds. All H atoms on C atoms have been omitted for clarity. [Symmetry codes: (i) *x* − 2, *y*, *z*; (ii) −*x* − 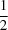, *y* + 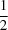, −*z* + 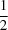; (iii) −*x*, −*y* + 2, −*z* + 1; (iv) −*x* − 1, −*y* + 1, −*z* + 1; (v) *x* − 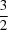, −*y* + 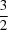, −*z* + 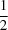; (vi) *x* − 2, *y* + 1, *z*; (vii) −*x* − 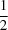, *y* + 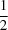, −*z* + 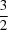; (viii) *x* − 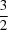, −*y* + 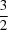, *z* − 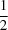; (ix) −*x* − 1, −*y* + 1, −*z* + 1; (x) −*x* − 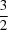, *y* + 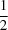, −*z* + 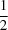; (xi) *x* − 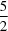, −*y* + 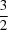, *z* − 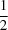; (xii) *x* − 2, *y* + 1, *z*; (xiii) *x* − 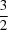, −*y* + 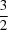, *z* + 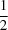.]

**Figure 7:**
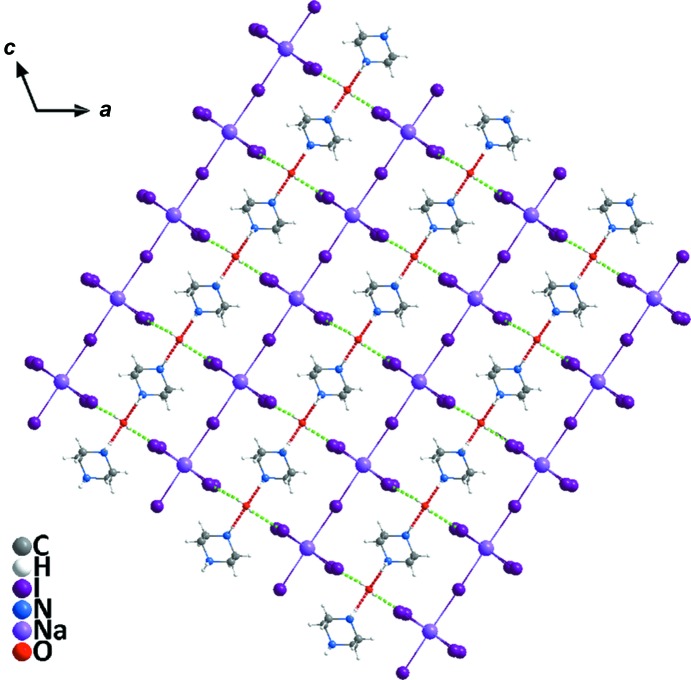
The hydrogen bonds of the O—H⋯I (green dashed lines) and N—H⋯O (red dashed lines) types in **2**, showing the two-dimensional network on the *ac* plane.

**Figure 8:**
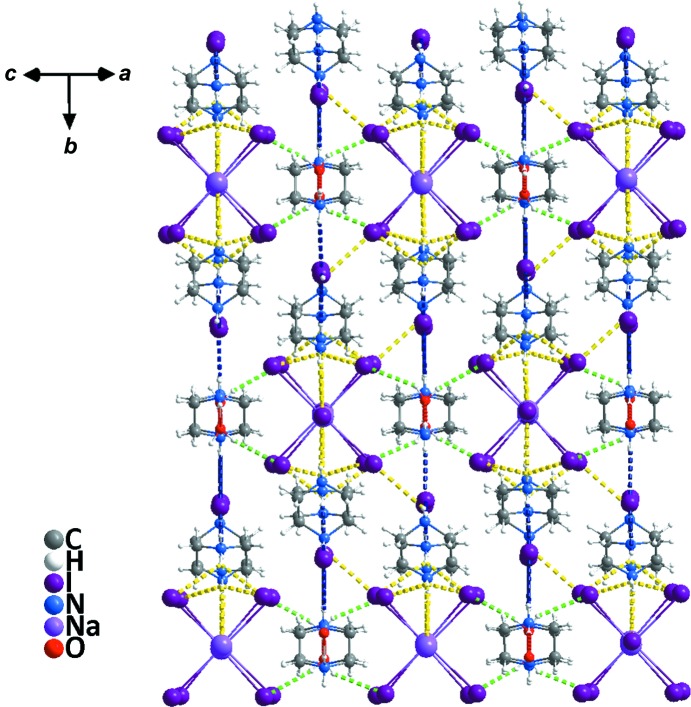
A packing view of compound **2**, showing the three-dimensional hydrogen-bonded network.

Compound **2** crystallizes in the monoclinic system (space group *P*2_1_/*n*) but displays a one-dimensional linear chain-like geometry. The asymmetry unit contains three whole piperazinediium cations, one lattice water mol­ecule, two dissociated iodide ions and one Na atom in a glide plane coordinated with five iodide ions. As can be seen from Fig. 5 ▸, each Na atom is coordinated by six I atoms, and two Na atoms are bridged by one I atom and extended in an infinite manner along a horizontal direction, thus presenting a one-dimensional linear chain. As shown in Table 4 ▸, the length of the Na—I bonds are within the reasonable range 3.180 (5)–3.515 (6) Å and the I—Na—I angles are in the ranges 84.32 (14)–94.56 (16) and 176.97 (18)–178.20 (17)°. It is worth noting that there are very complex hydrogen bonds in compound **2**. These hydrogen bonds can be divided roughly into four types (Fig. 6 ▸): (i) piperazinediium N atoms act as donors and water O atoms act as acceptors in N—H⋯O hydrogen bonds (red dashed lines); (ii) water O atoms act as donors and I atoms in the metal halide chain act as acceptors in O—H⋯I hydrogen bonds (green dashed lines); (iii) piperazinediium N atoms act as donors and bridging I atoms act as acceptors in N—H⋯I hydrogen bonds (yellow dashed lines); (iv) piperazinediium N atoms act as donors and the free I atoms act as acceptors in N—H⋯I hydrogen bonds (blue dashed lines). Detailed information of the hydrogen bonds is given in Table 5 ▸. As shown in Fig. 7 ▸, the water H atoms form hydrogen bonds with the I atoms on the two sides of the NaI_5_ chain (*i.e.* O1^i^—H1⋯I5^ii^ and O1^i^—H2⋯I2^ix^; Table 5 ▸), thus forming a two-dimensional network on the *ac* plane. On the other hand, the free I atoms (*i.e.* I6 and I7) and the bridging I atoms (*i.e.* I3) form N—H⋯I hydrogen bonds with the H atoms of the piperazinediium N atoms, which extends the two-dimensional network into a three-dimensional hydrogen-bonded supra­molecular network (Fig. 8 ▸).

## Summary   

Two new organic–inorganic hybrid sodium halides have been synthesized by adjusting the stoichiometric ratio of sodium iodide and piperazine. C_4_H_12_N_2_·NaI_3_, **1**, presents an inter­esting three-dimensional perovskite structure. However, compound 3C_4_H_12_N_2_·NaI_4_·3I·H_2_O, **2**, features a singular three-dimensional hydrogen-bonded network. The different structures of compounds **1** and **2** show that the stoichiometric ratio plays a key role in the synthesis of various frameworks.

## Supplementary Material

Crystal structure: contains datablock(s) C2C, C, global. DOI: 10.1107/S2053229618006885/qp3008sup1.cif

Structure factors: contains datablock(s) C2C. DOI: 10.1107/S2053229618006885/qp3008C2Csup2.hkl

Structure factors: contains datablock(s) C. DOI: 10.1107/S2053229618006885/qp3008Csup3.hkl

CCDC references: 1826738, 1826739

## Figures and Tables

**Table 1:** Experimental details

	**1**	**2**
Crystal data		
Chemical formula	(C_4_H_12_N_2_)[NaI_3_]	(C_4_H_12_N_2_)_3_[NaI_4_]I_3_·H_2_O
*M*_r_	491.85	1193.77
Crystal system, space group	Monoclinic, *C*2/*c*	Monoclinic, *P*2_1_/*n*
Temperature (K)	293	293
*a*, *b*, *c* (Å)	9.842 (6), 9.309 (6), 12.538 (8)	12.186 (2), 22.828 (5), 12.214 (2)
β (°)	93.450 (9)	111.89 (3)
*V* (Å^3^)	1146.6 (13)	3152.7 (12)
*Z*	4	4
Radiation type	Mo *K*α	Mo *K*α
μ (mm^−1^)	8.16	6.92
Crystal size (mm)	0.38 × 0.28 × 0.20	0.38 × 0.28 × 0.20

Data collection		
Diffractometer	Rigaku SCXmini	
Absorption correction	Multi-scan (*CrystalClear*; Rigaku, 2008 ▸)	
*T*_min_, *T*_max_	0.080, 0.195	0.112, 0.251
No. of measured, independent and observed [*I* > 2σ(*I*)] reflections	3288, 1311, 1153	20677, 7234, 4432
*R*_int_	0.083	0.075
(sin θ/λ)_max_ (Å^−1^)	0.648	0.649

Refinement		
*R*[*F* ^2^ > 2σ(*F* ^2^)], *wR*(*F* ^2^), *S*	0.058, 0.165, 1.03	0.085, 0.142, 1.09
No. of reflections	1311	7234
No. of parameters	48	252
No. of restraints	0	2
H-atom treatment	H-atom parameters constrained	H atoms treated by a mixture of independent and constrained refinement
Δρ_max_, Δρ_min_ (e Å^−3^)	1.80, −1.66	1.23, −1.05

Computer programs: *CrystalClear* (Rigaku, 2008 ▸), *SHELXS97* (Sheldrick, 2008 ▸), *SHELXL2014* (Sheldrick, 2015 ▸), *SHELXL2014* (Sheldrick, 2015 ▸) and *DIAMOND* (Brandenburg & Putz, 2005 ▸).

**Table 2:** Selected geometric parameters (Å, °) for **1**

C2—C1	1.504 (14)	Na1—I2	3.156 (2)
C2—N1^i^	1.532 (14)	Na1—I1	3.325 (5)
N1—C1	1.456 (13)		
			
Na1—I1—Na1^ii^	169.12 (12)	I2—Na1—I1^v^	84.40 (9)
Na1^iii^—I2—Na1	180.0	I2^iv^—Na1—I1^vi^	84.40 (9)
N1—C1—C2	111.6 (8)	I1^vi^—Na1—I1^v^	100.45 (19)
I2^iv^—Na1—I1	101.40 (10)	I1—Na1—I1^v^	169.12 (12)
I2—Na1—I1^iv^	101.40 (10)	I1^iv^—Na1—I1^vi^	169.12 (12)
I2^iv^—Na1—I2	166.6 (3)	I1^iv^—Na1—I1	86.15 (18)
I2—Na1—I1	88.41 (8)	I1^iv^—Na1—I1^v^	87.29 (5)
I2^iv^—Na1—I1^iv^	88.41 (8)	I1—Na1—I1^vi^	87.29 (5)
I2^iv^—Na1—I1^v^	87.06 (9)	C1—C2—N1^i^	108.4 (9)
I2—Na1—I1^vi^	87.06 (9)	C1—N1—C2^i^	110.2 (8)

Symmetry codes: (i) 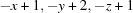; (ii) 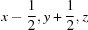; (iii) 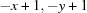, 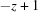; (iv) 
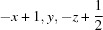
; (v) 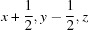; (vi) 
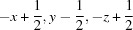
.

**Table 3:** Hydrogen-bond geometry (Å, °) for **1**

*D*—H⋯*A*	*D*—H	H⋯*A*	*D*⋯*A*	*D*—H⋯*A*
C1—H1*D*⋯I1^i^	0.97	3.12	3.937 (11)	143
C1—H1*C*⋯I2	0.97	3.23	3.914 (11)	129
C1—H1*C*⋯I1^vii^	0.97	3.14	3.790 (10)	126
C2—H2*B*⋯I1^viii^	0.97	3.17	3.930 (11)	136
C2—H2*A*⋯I1^iv^	0.97	3.23	3.930 (13)	131
N1—H1*B*⋯I1^i^	0.89	2.80	3.628 (10)	156
N1—H1*A*⋯I2^ii^	0.89	3.11	3.677 (8)	123
N1—H1*A*⋯I1^vii^	0.89	3.14	3.746 (8)	127

Symmetry codes: (i) 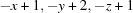; (ii) 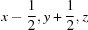; (iv) 
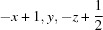
; (vii) 
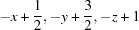
; (viii) 
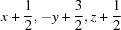
.

**Table 4:** Selected geometric parameters (Å, °) for **2**

C1—N1	1.515 (14)	C9—C10	1.480 (18)
C1—C2	1.511 (18)	C10—N6	1.477 (13)
C2—N2	1.451 (16)	C11—C12	1.514 (17)
C3—N1	1.481 (14)	C11—N5	1.487 (13)
C3—C4	1.519 (16)	C12—N6	1.509 (13)
C4—N2	1.447 (17)	I1—Na1	3.419 (6)
C5—C6	1.527 (16)	I2—Na1	3.205 (5)
C5—N3	1.472 (13)	I3—Na1	3.381 (5)
C6—N4	1.452 (14)	I3—Na1^i^	3.456 (5)
C7—C8	1.532 (16)	I4—Na1	3.515 (6)
C7—N3	1.475 (13)	I5—Na1	3.180 (5)
C8—N4	1.486 (14)	Na1—I3^ii^	3.456 (5)
C9—N5	1.493 (14)		
			
C10—N6—C12	111.1 (9)	I3—Na1—I1	91.38 (13)
C10—C9—N5	110.8 (10)	I5—Na1—I4	94.12 (14)
C11—N5—C9	110.5 (8)	I5—Na1—I3^ii^	92.63 (13)
C2—C1—N1	111.0 (10)	I5—Na1—I1	86.95 (13)
C3—N1—C1	109.5 (10)	I5—Na1—I3	88.73 (12)
C4—N2—C2	114.6 (12)	I5—Na1—I2	177.4 (2)
C5—N3—C7	112.7 (10)	N1—C3—C4	113.1 (10)
C6—N4—C8	111.8 (10)	N2—C4—C3	109.0 (11)
I1—Na1—I4	178.20 (17)	N2—C2—C1	108.3 (11)
I1—Na1—I3^ii^	91.40 (13)	N3—C7—C8	110.8 (10)
I2—Na1—I4	84.32 (12)	N3—C5—C6	110.7 (10)
I2—Na1—I3^ii^	89.46 (12)	N4—C8—C7	105.3 (11)
I2—Na1—I1	94.56 (14)	N4—C6—C5	106.4 (10)
I2—Na1—I3	89.12 (13)	N5—C11—C12	111.3 (10)
I3^ii^—Na1—I4	90.00 (12)	N6—C12—C11	109.0 (10)
I3—Na1—I4	87.19 (13)	N6—C10—C9	110.8 (10)
I3—Na1—I3^ii^	176.97 (18)	Na1—I3—Na1^i^	176.87 (7)

Symmetry codes: (i) 
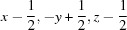
; (ii) 
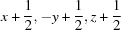
.

**Table 5:** Hydrogen-bond geometry (Å, °) for **2**

*D*—H⋯*A*	*D*—H	H⋯*A*	*D*⋯*A*	*D*—H⋯*A*
N6^vi^—H6*B*⋯I6^vi^	0.89	2.99	3.626 (11)	130
N6^vi^—H6*A*⋯O1^iii^	0.89	1.97	2.856 (16)	175
N5^vi^—H5*B*⋯O1^ii^	0.89	1.99	2.878 (15)	178
N5^vi^—H5*A*⋯I7^xii^	0.89	2.83	3.557 (10)	140
N4^v^—H4*B*⋯I6^xiii^	0.89	2.55	3.440 (12)	175
N4^v^—H4*A*⋯I5^vii^	0.89	2.99	3.663 (13)	134
N4^v^—H4*A*⋯I1^vii^	0.89	3.25	3.881 (14)	130
N3^v^—H3*B*⋯I4^iv^	0.89	3.22	3.748 (11)	121
N3^v^—H3*B*⋯I3^ix^	0.89	3.22	3.767 (12)	122
N3^v^—H3*B*⋯I2^ix^	0.89	3.04	3.610 (11)	124
N3^v^—H3*A*⋯I7^xii^	0.89	2.68	3.543 (12)	165
N2^iv^—H2*B*⋯I2^ix^	0.89	3.14	3.867 (16)	140
N2^iv^—H2*A*⋯I3^ix^	0.89	2.70	3.405 (13)	138
N1^iv^—H1*B*⋯I6^xi^	0.89	2.62	3.496 (11)	169
N1^iv^—H1*A*⋯I7^xii^	0.89	2.92	3.613 (11)	136
N1^iv^—H1*A*⋯I1^viii^	0.89	3.32	3.804 (11)	117
O1^i^—H2⋯I2^ix^	0.85 (1)	2.68 (9)	3.471 (12)	155 (18)
O1^i^—H1⋯I5^ii^	0.85 (1)	2.69 (4)	3.501 (11)	161 (11)

Symmetry codes: (i) 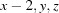; (ii) 
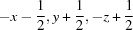
; (iii) 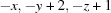; (iv) 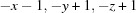; (v) 
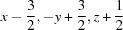
; (vi) 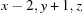; (vii) 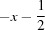, 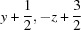; (viii) 
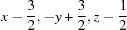
; (ix) 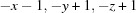; (x) 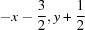, 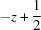; (xi) 
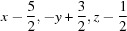
; (xii) 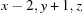; (xiii) 
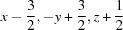
.

## References

[bb1] Brandenburg, K. & Putz, H. (2005). *DIAMOND*. Crystal Impact GbR, Bonn, Germany.

[bb2] Galkowski, K., Mitioglu, A., Miyata, A., Plochocka, P., Portugall, O., Eperon, G. E., Wang, J. T. W., Stergiopoulos, T., Stranks, S. D., Snaith, H. J. & Nicholas, R. J. (2016). *Energy Environ. Sci.* **9**, 962–970.

[bb3] Glazer, A. M. (1972). *Acta Cryst.* B**28**, 3384–3392.

[bb4] Glazer, A. M. (1975). *Acta Cryst.* A**31**, 756–762.

[bb5] Jin, H. H., Sang, H. I., Noh, J. H., Mandal, T. N., Lim, C. S., Chang, J. A., Yong, H. L., Kim, H. J., Sarkar, A. & Nazeeruddin, M. K. (2009). *Nat. Photonics*, **7**, 486–491.

[bb6] Jodlowski, A. D., Yépez, A., Luque, R., Camacho, L. & De, M. G. (2016). *Angew. Chem. Int. Ed.* **55**, 14972–14977. 10.1002/anie.201607397 27791299

[bb7] Lee, Y., Mitzi, D. B., Barnes, P. W. & Vogt, T. (2003). *Phys. Rev. B*, **68**, 366–369.

[bb8] Liao, W. Q., Tang, Y. Y., Li, P. F., You, Y. M. & Xiong, R. G. (2017). *J. Am. Chem. Soc.* **139**, 18071–18077. 10.1021/jacs.7b10449 29144132

[bb9] Liao, W. Q., Zhao, D. W., Yu, Y., Grice, C. R., Wang, C. L., Cimaroli, A. J., Schulz, P., Meng, W. W., Zhu, K., Xiong, R. G. & Yan, Y. Y. (2016*a*). *Adv. Mater.* **28**, 9333–9340. 10.1002/adma.201602992 27571446

[bb10] Liao, W. Q., Zhao, D. W., Yu, Y., Shrestha, N., Ghimire, K., Grice, C. R., Wang, C. L., Xiao, Y. Q., Cimaroli, A. J., Eiiingson, R. J., Podraza, N. J., Zhu, K., Xiong, R. G. & Yan, Y. Y. (2016*b*). *J. Am. Chem. Soc.* **138**, 12360–12363. 10.1021/jacs.6b08337 27622903

[bb11] Pan, Q., Liu, Z. B., Tang, Y. Y., Li, P. F., Ma, R. W., Wei, R. Y., Zhang, Y., You, Y. M., Ye, H. Y. & Xiong, R. G. (2017). *J. Am. Chem. Soc.* **139**, 3954–3957. 10.1021/jacs.7b00492 28248096

[bb12] Paton, L. A. & Harrison, W. T. (2010). *Angew. Chem. Int. Ed.* **49**, 7850–7853.

[bb13] Rigaku (2008). *CrystalClear*. Rigaku Corporation, Tokyo, Japan.

[bb14] Saparov, B. & Mitzi, D. B. (2016). *Chem. Rev.* **116**, 4558–4596. 10.1021/acs.chemrev.5b00715 27040120

[bb15] Sheldrick, G. M. (2008). *Acta Cryst.* A**64**, 112–122. 10.1107/S0108767307043930 18156677

[bb16] Sheldrick, G. M. (2015). *Acta Cryst.* C**71**, 3–8.

[bb17] Shi, Z., Guo, J., Chen, Y., Li, Q., Pan, Y., Zhang, H., Xia, Y. & Huang, W. (2017). *Adv. Mater.* **29**, 1605005–1605033. 10.1002/adma.201605005 28160346

[bb18] Veldhuis, S. A., Boix, P. P., Yantara, N., Li, M., Sum, T. C., Mathews, N. & Mhaisalkar, S. G. (2016). *Adv. Mater.* **28**, 6804–6834. 10.1002/adma.201600669 27214091

[bb19] Xu, W. J., Li, P. F., Tang, Y. Y., Zhang, W. X., Xiong, R. G. & Chen, X. M. (2017). *J. Am. Chem. Soc.* **139**, 6369–6375. 10.1021/jacs.7b01334 28402110

[bb20] Yang, W. S., Noh, J. H., Jeon, N. J., Kim, Y. C., Ryu, S., Seo, J. & Seok, S. I. (2015). *Science*, **348**, 1234–1237. 10.1126/science.aaa9272 25999372

[bb21] You, Y. M., Liao, W. Q., Zhao, D., Ye, H. Y., Zhang, Y., Zhou, Q., Niu, X., Wang, J., Li, P. F., Fu, D. W., Wang, Z., Gao, S., Yang, K., Liu, J. M., Li, J., Yan, Y. & Xiong, R. G. (2017). *Science*, **357**, 306–309. 10.1126/science.aai8535 28729511

[bb22] Zhang, W. Y., Tang, Y. Y., Li, P. F., Shi, P. P., Liao, W. Q., Fu, D. W., Ye, H. Y., Zhang, Y. & Xiong, R. G. (2017). *J. Am. Chem. Soc.* **139**, 10897–10902. 10.1021/jacs.7b06013 28719192

